# The Functioning of a Cortex without Layers

**DOI:** 10.3389/fnana.2017.00054

**Published:** 2017-07-12

**Authors:** Julien Guy, Jochen F. Staiger

**Affiliations:** ^1^Institute for Neuroanatomy, University Medical Center Göttingen, Georg-August-University Göttingen, Germany; ^2^DFG Center for Nanoscale Microscopy and Molecular Physiology of the Brain (CNMPB) Göttingen, Germany

**Keywords:** neocortex, cortical circuits, reeler mutant mouse, developmental plasticity, optogenetics, lemniscal pathway

## Abstract

A major hallmark of cortical organization is the existence of a variable number of layers, i.e., sheets of neurons stacked on top of each other, in which neurons have certain commonalities. However, even for the neocortex, variable numbers of layers have been described and it is just a convention to distinguish six layers from each other. Whether cortical layers are a structural epiphenomenon caused by developmental dynamics or represent a functionally important modularization of cortical computation is still unknown. Here we present our insights from the reeler mutant mouse, a model for a developmental, “molecular lesion”-induced loss of cortical layering that could serve as ground truth of what an intact layering adds to the cortex in terms of functionality. We could demonstrate that the reeler neocortex shows no inversion of cortical layers but rather a severe disorganization that in the primary somatosensory cortex leads to the complete loss of layers. Nevertheless, the somatosensory system is well organized. When exploring an enriched environment with specific sets of whiskers, activity-dependent gene expression takes place in the corresponding modules. Precise whisker stimuli lead to the functional activation of somatotopically organized barrel columns as visualized by intrinsic signal optical imaging. Similar results were obtained in the reeler visual system. When analyzing pathways that could be responsible for preservation of tactile perception, lemniscal thalamic projections were found to be largely intact, despite the smearing of target neurons across the cortical mantle. However, with optogenetic experiments we found evidence for a mild dispersion of thalamic synapse targeting on layer IV-spiny stellate cells, together with a general weakening in thalamocortical input strength. This weakening of thalamic inputs was compensated by intracortical mechanisms involving increased recurrent excitation and/or reduced feedforward inhibition. In conclusion, a layer loss so far only led to the detection of subtle defects in sensory processing by reeler mice. This argues in favor of a view in which cortical layers are not an essential component for *basic* perception and cognition. A view also supported by recent studies in birds, which can have remarkable cognitive capacities despite the lack of a neocortex with multiple cortical layers. In conclusion, we suggest that future studies directed toward understanding cortical functions should rather focus on circuits specified by functional cell type composition than mere laminar location.

## The Concept of Cortical Layers

In 1867, Theodor Meynert, the father of cytoarchitectonics, published an account of his microscopic examinations of the mammalian cerebral cortex, the first to propose a subdivision of the cortex in layers based on cellular composition (Meynert, 1867). The concept of cortical layers was not unknown until this point, and had in fact emerged from observations of the cortex by naked eye (Gennari, 1784; Vicq d’Azyr, 1786; Baillarger, 1840). Its gradual historical development is characterized by a great variation, from three to nine, in the number of layers proposed (Meynert, 1867). The practice of defining layering by cellular composition, however, has endured to this day, and largely contributed to our contemporary view of the functional organization of the neocortex. This view subdivides the neocortex into six layers, defined by the morphological cell types they are composed of, their connectivity, developmental origins and patterns of gene expression. This consensus, although fairly well established, is still the subject of ongoing refinements (Zilles and Wree, 1995; Skoglund et al., 1997; Lein et al., 2007; Feldmeyer, 2012; Staiger, 2015; Staiger et al., 2015).

An anatomical description of cortical lamination can hardly ignore the question of laminar function. The fact that cortical layers are composed of distinct neuron types with unique properties and specific connectivity is suggestive of a division of labor among them, whereby each layer carries a fraction of the computational load of a column. For example, a common view is that information is processed in a sequential or feedforward manner in the cortical column, each layer completing its own computation before passing the outcome to the next along the canonical microcircuit. Thus, in the words of Kenneth D. Miller: “*in order to understand the computations being performed by the cortex, we need to understand the nature of the processing undertaken by each layer*” (Miller et al., 2001).

## What Would be A Suitable Description of The Function of each Layer?

Ideally, a function that identifies what operations are performed exclusively within one layer, as opposed to operations emerging from the collective action of multiple layers. For example, a recurring statement in the literature considers layer IV of the rodent somatosensory cortex as the main input stage for sensory information, due to its dense innervation by the ventral posterior medial nucleus of the thalamus (Chmielowska et al., 1989; Staiger et al., 1996; Wimmer et al., 2010; Oberlaender et al., 2012) and its vigorous, short latency responses to whisker touch (Simons, 1978; Armstrong-James and Fox, 1987). An imaginary description of the role of individual layers in sensory neocortex could use similar terms: layer IV of the cortex acts as a relay and an amplifier of sensory information (due to the dense reciprocal connections among thalamorecipient excitatory neurons; Feldmeyer et al., 1999; Schubert et al., 2003), perhaps adapting the gain of amplification to behavioral requirements. Layer II/III receives tuned sensory information from layer IV and weaves it together with contextual information (provided by associational cortico-cortical input) to produce the first percept of the external object. Layer II/III informs layer V of the percept; due to its many long range outputs, layer V broadcasts the content of the percept to various locations within the brain, and thus conjures up relevant memories associated with it but stored elsewhere in the cortex. Layer VI, finally, signals back to the sensory thalamus that the ongoing cortical calculation has ended. Although the functions listed here are fictional, an adequate description of laminar function would be of that kind (Schubert et al., 2007; Harris and Mrsic-Flogel, 2013; Harris and Shepherd, 2015).

However desirable it may be, the emergence of such a model has been frustrated by the lack of suitable experimental approaches. Indeed, one can hardly conceive of a reversible surgical or pharmacological inactivation of one layer that would spare all others, although some attempts have been made (Huang et al., 1998; Fox et al., 2003; Wright and Fox, 2010; Constantinople and Bruno, 2013). Similarly, the advent of optogenetics is of limited help here. Because one layer’s output is the other’s input, reversible optogenetic inactivation of one layer would eventually compromise computation in the entire network, making it difficult to isolate the role of the inactivated layer in generating behavior. In order to determine whether layers are involved in cortical computations *at all*, the field would rather benefit from comparing model organisms possessing laminated vs. non laminated cortices. Ideally, such models would belong to the same species and be identical in all respects expect cortical lamination. The cortices or brain areas to be compared should be composed of similar neurons, forming identical networks performing the same functions.

## The *Reeler* Neocortex as A Model System

**Figure 1:**
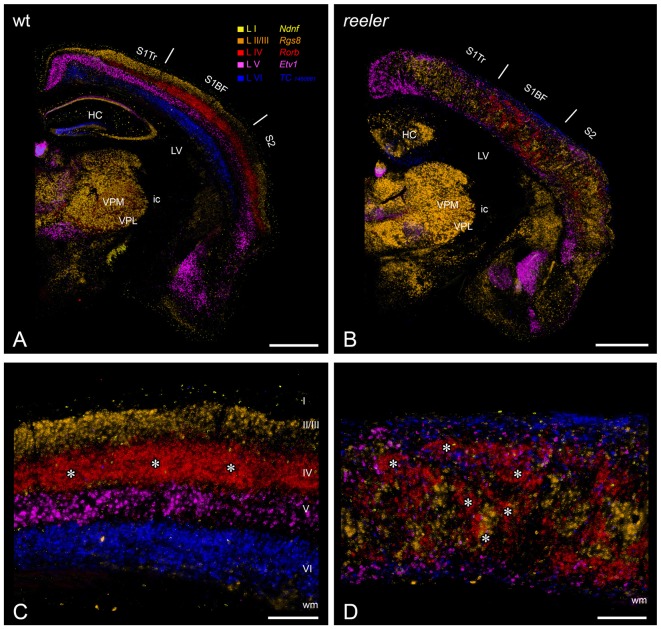
Laminar fate markers show the dramatic disorganization of cortical layers in the reeler brain. **(A,B)** Coronal hemisections through the brain of a wild type **(A)** and a reeler mouse **(B)**, at the level of the primary somatosensory (barrel) cortex. Laminar fate markers (labeled in **A**) have been stained by *in situ*-hybridization in serial sections, false color-coded and overlaid to obtain a comprehensive impression (modified from Wagener et al., 2016). The overview shows that the general anatomical layout of the brain in terms of subcortical nuclei and cortical areas is basically normal. **(C,D)** Higher magnification through the barrel cortex shows a typical layering of a granular cortex **(C)**, with barrel-like clustered L IV-spiny stellates (*asterisks*). In the reeler mutant **(D)**, most cell types can be found anywhere across the cortical depth, with L IV-fated cells also forming cluster, which we called barrel equivalents (*asterisks*). *Roman numerals* mark cortical layers. Abbreviations: HC, hippocampus; ic, internal capsule; LV, lateral ventricle; S1BF, barrel field of the primary somatosensory cortex; S1Tr, trunk region of the primary somatosensory cortex; S2, secondary somatosensory cortex; VPL, nucleus ventralis posterolateralis; VPM, nucleus ventralis posteromedialis. Scale bars: **(A,B)**—1000 μm; **(C,D)**—250 μm.

In our opinion, the reeler mutant mouse provides the closest approximation to such a model. The mutation was first documented after it appeared spontaneously at the Institute of Animal Genetics in Edinburgh and results in the loss of expression of the reelin protein (Curran and D’Arcangelo, 1998; Tissir and Goffinet, 2003). This large extracellular protein is expressed by Cajal-Retzius-cells during cortical development (Frotscher et al., 2009). Through signaling via its membrane receptors ApoEr2 and VLDLr (Bock and May, 2016), reelin guides the migration of newborn neurons and orchestrates the development of cortical layers. In the absence of reelin or its receptors, the process of neuronal migration is compromised, which causes severe abnormalities in cortical lamination. The resulting phenotype was initially described as an inversion of the layers, whereby the normal “inside out” pattern was inverted into an “outside in” pattern. There, layer VI becomes situated below the pia (forming the so called superplate by merging with the marginal zone, representing prospective layer I) and layer II above the white matter (Caviness and Sidman, 1973; Caviness et al., 1988). More recent studies, however, have revealed a far more disorganized pattern (Figure 1), where cortical neurons are intermingled in a chaotic manner irrespective of cortical depth, forming patterns which surprisingly seem to vary according to cortical area (Dekimoto et al., 2010; Wagener et al., 2010; Boyle et al., 2011; Pielecka-Fortuna et al., 2015).

How well does the reeler mouse fit as a model with the requirements listed above? The neocortex, hippocampus and cerebellum, the brain structures affected most by reelin deficiency, have received considerable attention over the years. A repeated finding was that all cell types normally found in these regions were all present in reeler (Caviness and Sidman, 1973; Stanfield and Cowan, 1979). The total number of neurons populating the reeler or the wild type cortex is roughly equivalent, although late born (supragranular) neurons are somewhat overrepresented at the expense of early born (infragranular) neurons (Polleux et al., 1998; Wagener et al., 2010, 2016; Boyle et al., 2011). The relative numbers of excitatory and inhibitory neurons are also unchanged (Hevner et al., 2004; Wagener et al., 2016). Furthermore, neurons appear to retain their correct properties despite their ectopic positions. Molecular markers typically expressed in a layer-specific fashion are still expressed by displaced neurons (Katsuyama and Terashima, 2009; Boyle et al., 2011; Wagener et al., 2016), suggesting that their molecular identity is not compromised by the lack of lamination. The morphology of defined neuronal types has been investigated in some detail and is relatively unchanged (Guy et al., 2016), although some excitatory types see a reduction in the number of dendritic spines (Niu et al., 2008), and some inhibitory types have longer dendrites with more branches (Yabut et al., 2007).

**Figure 2:**
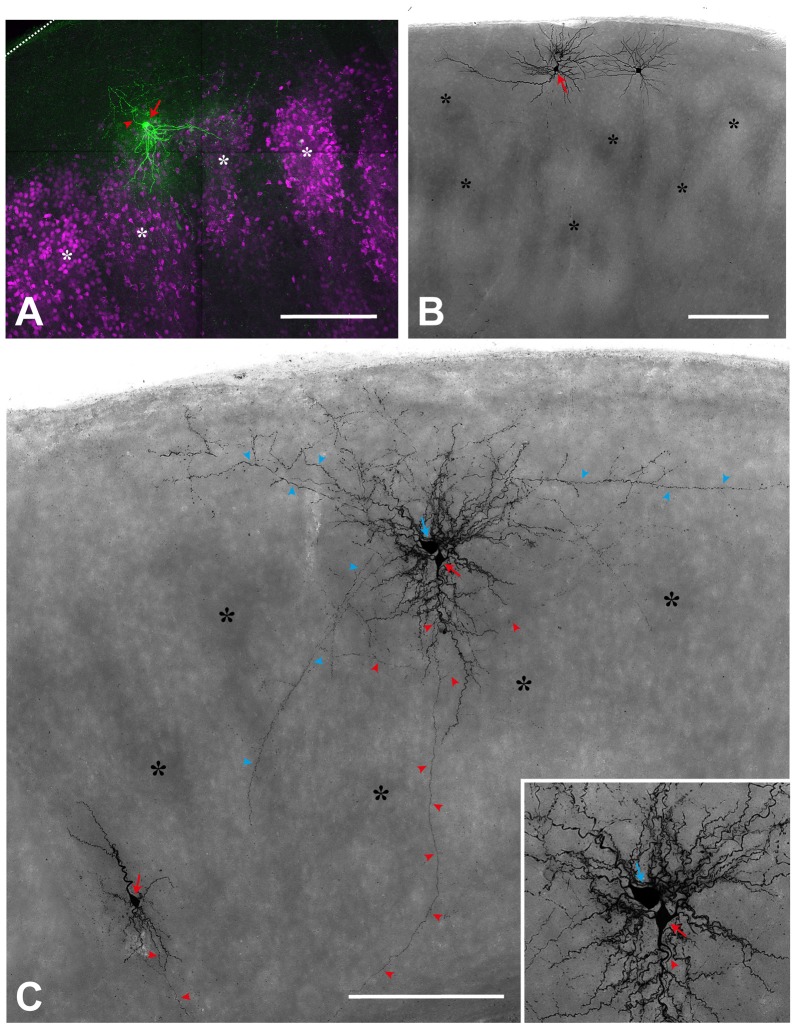
Single cell fillings show the aberrant morphology of several types in the reeler cortex. **(A)** Regular-spiking spiny stellate cell (*red arrow*), located on top of two barrel equivalents (*asterisks*; labeled by Sccn1a-cre/tdTomato; see Guy et al., 2015, 2016). Please note that the dendrites spread out in a V-shaped manner to reach to neighboring cell clusters whereas the axon (*red arrowhead*) initially is directed toward the pial surface (*dashed line*). **(B)** Repetitive-bursting (probably “L Vb”) pyramidal cell (*red arrow*) with a horizontally-oriented apical dendrite. **(C)** Cortical slice, in which three neurons have been recorded and labeled. A small up-right, regular-spiking pyramidal cell (*red arrow*; lower left), whose axon (*red arrowheads*) is directed toward the white matter. An inverted regular-spiking pyramidal cell (*red arrow*; see inset for details) shows an axon (*red arrowheads*) originating from the apical dendrite, which points toward the white matter). Directly next to it, a fast-spiking large basket cell (*blue arrow*) is labeled, whose axon (*blue arrowheads*), in addition to many local collaterals, issues divergent axonal projections. Scale bars: **(A)**—100 μm; **(B,C)**—250 μm.

One oddity found in reeler is an apparent distortion in the dendritic arbors of some of the ectopic neurons (Figure 2). For instance, the apical dendrites of large pyramidal neurons may travel in an oblique fashion towards the pia, they may orient themselves horizontally, and even be inverted (Landrieu and Goffinet, 1981; Simmons et al., 1982; Terashima et al., 1983, 1985; Silva et al., 1991). Similarly distorted dendritic arbors were reported in the hippocampus (Stanfield and Cowan, 1979) and the cerebellum (Heckroth et al., 1989). However, these are better explained by the fact that ectopic cells may find themselves outside of their home structure, where space constraints makes a normal orientation impossible, rather than by an abnormality in their intrinsic morphology—especially in the cerebral cortex. An alternative explanation is that these disorientations result from the attempt of dendritic outgrowth mechanisms to sample from their correct afferent pathways (Pinto Lord and Caviness, 1979), which can be distorted in bizarre manners, best exemplified by lemniscal thalamic projections to the neocortex. The thick myelinated fibers first ascend in an oblique manner to the pial surface before abruptly turning and re-entering the cortical plate where they branch into their terminal arborizations (Caviness and Frost, 1983; Pielecka-Fortuna et al., 2015; Wagener et al., 2016), a phenomenon which has also been observed with *in vivo*-fiber tracking (Harsan et al., 2013). This fiber trajectory could be caused by transient synapses that thalamic synapses form with subplate neurons, which in reeler mice are found just below the pia in the superplate (Higashi et al., 2005). Of course one may wonder whether these morphologically aberrant reeler neurons have also aberrant electrophysiological properties. However, what little evidence exists shows that differences are slim: neurons in the neocortex and the hippocampus retain normal firing patterns and most other intrinsic properties (Silva et al., 1991; Kowalski et al., 2010; Guy et al., 2016).

Neuronal connectivity has also been the subject of scrutiny, and was repeatedly found to be largely intact in reeler (Caviness and Rakic, 1978). Indeed, although thalamocortical fibers follow an unorthodox trajectory through the cortex, they are capable of finding their target areas and cells, especially the layer IV equivalent neurons, in spite of their ectopic positions (Steindler and Colwell, 1976; Terashima et al., 1987; Wagener et al., 2016). Cortico-cortical connectivity is preserved as well in the somatosensory (Guy et al., 2015) and visual cortex (Lemmon and Pearlman, 1981; Simmons et al., 1982). Interhemispheric connections are established in a normal pattern as well (Caviness and Yorke, 1976). Finally, efferent connectivity is also preserved, as shown in the piriform and motor cortices (Terashima et al., 1987; Diodato et al., 2016). Overall, it appears that the mutant and normal cortex are composed of the same elements forming virtually identical circuits. Thus, their main difference resides in the absence of lamination characteristic of the reeler phenotype, making this mutant a fitting model for our endeavor.

## Functional Phenotype of The *Reeler* Mouse

So what are the functional consequences of a lack of cortical lamination in the reeler mouse? In line with the largely normal connectivity in mutant mice, most studies found little difference in various measures of cortical function. On a circuit level, based on c-fos expression as well as intrinsic signal optical imaging, our group found normal responses to tactile stimulation in the reeler somatosensory cortex as well as in the corresponding subcortical relay stations (Guy et al., 2015; Wagener et al., 2016). Although it had already been shown that some kind of deviant barrels form in the somatosensory cortex of reeler (Caviness et al., 1976; Welt and Steindler, 1977), we also demonstrated that the barrel field retains its proper somatotopic organization, a rather surprising finding in the light of the massive lamination defects (Wagener et al., 2010; Guy et al., 2015). A study using similar approaches found comparable results in the visual cortex, where retinotopic organization and normal visually-evoked responses were observed (Pielecka-Fortuna et al., 2015). The first electrophysiological study of the reeler brain recorded local field potentials in the mutant hippocampus *in vitro*, and concluded that perforant path input to granule cells, as well as Schaffer collateral input to CA1 pyramidal cells were functional (Bliss and Chung, 1974). The response properties of individual neurons have been investigated as well. Still in the hippocampus *in vitro*, Kowalski et al. (2010) showed that mossy cells of the hilar region of the dentate gyrus receive direct input from granule cells in both mutant and normal mice. Using channelrhodopsin expression to control thalamocortical fiber activity and whole cell recordings *in vitro*, our group provided evidence that spiny stellate neurons, a main constituent of barrels in the somatosensory cortex, receive strong direct input from the ventral posteromedial nucleus in reeler, as they do in normal animals (Guy et al., 2016; Wagener et al., 2016). A few studies investigated the receptive field properties of individual neurons in the reeler visual cortex with single unit, extracellular recordings. Beyond the fact that neurons in the visual cortex respond to various sensory stimuli in the anesthetized reeler mouse, one such study discovered that ocular dominance was largely preserved in the mutant, with similar proportion of cells responding to contra- or ipsilateral stimulation (Dräger, 1981; Simmons and Pearlman, 1983). In addition, in these studies, normal receptive field types were observed in the reeler cortex: both oriented and non-oriented receptive fields, as well as simple and complex receptive fields, although in somewhat changed relative proportions, with a higher fraction of non-oriented neurons in the mutant. Another noteworthy peculiarity of the mutant visual cortex is its higher proportion of neurons showing very broad receptive fields (Dräger, 1981; Lemmon and Pearlman, 1981). These apparent abnormalities could however be due to differences in the cell populations sampled, as cortical depth is a poor predictor of the cell type recorded in reeler. Overall, physiological responses to sensory stimulation appear largely preserved in the absence of cortical layers, a good indication that functional connectivity is mostly unchanged.

## Behavioral Performance of The *Reeler* Mutant Mouse

A predictable consequence of unaltered functional connectivity is that reeler performs well in tests of perceptual or mnesic capacities. Alas, a hallmark of the reeler phenotype is a severe ataxia (Falconer, 1951; Magdaleno et al., 2002), linked to a well described cerebellar atrophy (Badea et al., 2007), accompanied by cell loss and dispersion (Mariani et al., 1977). Together with the high mortality rate within weeks of birth due to impaired feeding after weaning, this motor impairment has somewhat deterred attempts at investigating behavioral anomalies in the mutant, as many behavioral tests rely on a motor readout. What literature exists is well aligned with our expectation, however. Early observations have reported that reeler mice display a wide and overall normal range of behaviors once adult, including mating (Myers, 1970), in spite of notable delays in sensorimotor and social development (Romano et al., 2013). In what is probably the broadest behavioral study of reeler to date, Salinger et al. (2003) reported that mutant mice can use olfactory cues to find a hidden food pellet; in a separate assay, they were found to have normal depth perception; acoustic responsiveness was found unchanged as well. Although the study reported anomalies in social behavior and reduced anxiety levels, it concluded that sensory function is normal in reeler. Our group has reported that mutant mice normally use their whiskers to explore a novel enriched environment in the dark (Wagener et al., 2010), suggesting proper sensorimotor function. More detailed studies of visual prowess have examined the optokinetic nystagm, the reflex by which mice make head movements to follow a drifting grating. No impairment was found in the mutant either in visual acuity or in their sensitivity to the contrast and spatial frequency of the grating used (Sinex et al., 1979; Pielecka-Fortuna et al., 2015), suggesting that basic visual function is intact as well. On the basis of this largely preserved perception, reeler animals exhibit not only spontaneous exploratory behavior but also seem capable of spatial learning. Goldowitz and Koch (1986) tested the ability of several neurological mutants to learn an 8-arm-radial maze; although the initial performance of reeler was poorer than normal mice, it was equalized by training. Similar results were obtained using a visual water task, in which mice swim to a submerged platform signaled by an oriented grating. Reeler animals were able to learn the task at the same pace as wild type controls, and could recall the task months after the initial training (Pielecka-Fortuna et al., 2015). These results suggest that at least the basic function of sensory cortex and hippocampus is spared. In summary, perception, learning and memory are largely unaffected in the mutant mouse, and although some behavioral anomalies were reported, they seem to relate to social and emotional function rather than sensory acuity (Salinger et al., 2003).

## A Puzzling Conclusion: Layers have No Apparent Function

**Figure 3:**
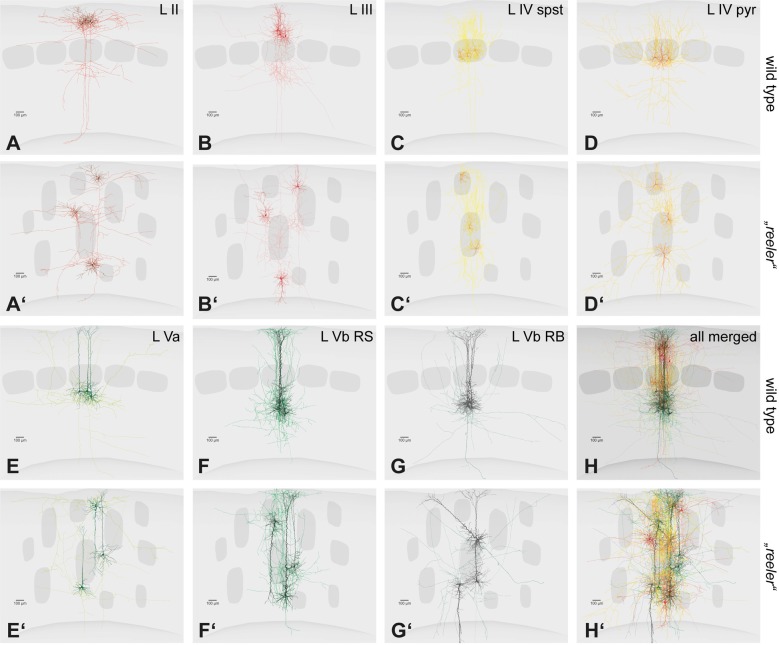
Single cell fillings of excitatory neurons in the wild type and their hypothetical counterparts in the reeler cortex. **(A–H)** Principal cells of the barrel cortex (*n* = 3 overlaid for each type) show typical layer-dependent organization of their dendritic and axonal arbors. Original data published (Schubert et al., 2003, 2006; Staiger et al., 2004, 2015, 2016). **(A′–H′)** Hypothetical schemes showing how reeler equivalent cells could be organized, after rotating and re-distributing them over the cortical depth. *Roman numerals* mark cortical layers. Abbreviations: pyr, pyramidal cell; spst, spiny stellate cell; RB, repetitive-bursting; RS, regular-spiking.

Although much of the reeler brain morphology, physiology and behavior remains to be documented, the evidence briefly summarized here is sufficient to form an opinion as to whether or not cortical layers have a function. First, the cortex of the reeler mouse houses “normal” cell types, with their properties mostly unchanged. Second, even though they are ectopic, these neurons form appropriate connections and networks, which is difficult to envisage given the substantial deviation from normal of many neurons types (Figure 3). Third, both individual cells and networks respond to sensory input in a seemingly normal way. Fourth, perception, memory and overall behavior are not obviously compromised. It would thus appear that the loss of cortical lamination does not impair cortical function in any recognizable way, and that layers are in fact completely expendable. In other words, we hold the view that *layers as such have no function* in the context of information processing, although we do not exclude that they may serve different purposes. It follows that asking oneself what the role of an individual layer is, in terms of its share of the total computational workload, is misguided. This does not rule out other, supportive roles for layers, for example that they organize neurons into modules in which computation can be run at a lesser metabolic cost. These will be discussed below, after a cautionary note about the reeler mouse and an excursion to another model.

## Limitations of The Reeler Model

Even though the idea that layers have no computational functions is seductive in its simplicity, we must in all fairness acknowledge that the reeler model has limitations that must be mentioned. For one, a few misconnections have been reported in brains areas beyond the neocortex. In the cerebellum of reeler, abnormal synapses were found between mossy fibers and Purkinje cell spines (Mariani et al., 1977; Wilson et al., 1981). In the hippocampus, Kowalski et al. (2010) found aberrant input from the perforant path to mossy cells. Granted, no example of aberrant input was discovered in the neocortex but their existence has been hypothesized (Caviness and Rakic, 1978) and if confirmed, would mean that the reeler model no longer fits the requirement of network equivalence. In addition, reelin expression persists after birth in a heterogeneous subset of GABAergic interneurons (Alcántara et al., 1998; Pohlkamp et al., 2014). The roles of reelin in the adult brain are thought to be multiple and the subject of ongoing research, but some bear potentially significant consequences for our argument. In particular, the protein has been shown to modulate synaptic transmission by various mechanisms. Postsynaptically, reelin mediates an enhancement of Ca^2+^ currents through the NMDA receptor (Chen et al., 2005), and increases AMPA receptor integration in the plasma membrane (Qiu et al., 2006b). Presynaptically, the absence of reelin alters the composition of the SNARE complex and the number of vesicles at hippocampal synapses, an effect accompanied by a decrease in paired pulse facilitation (Hellwig et al., 2011). In line with this role in synaptic transmission, reelin was also shown to enhance hippocampal long-term potentiation (Beffert et al., 2005; Qiu et al., 2006b). In addition, evidence is mounting that GABAergic transmission is weakened by a loss of reelin, resulting in a shift in the excitation-inhibition balance with potentially far-reaching consequences (Qiu et al., 2006a; Guy et al., 2016; Bouamrane et al., 2017). Finally, at least one study reported a slight anomaly in visual perception in reeler mice, namely impairment in orientation discrimination (Pielecka-Fortuna et al., 2015).

Taken together, these results reveal a conundrum: should perceptual or behavioral anomalies be discovered in reeler, how to attribute them to the loss of layers or to abnormalities in synaptic transmission? This problem will predictably limit how much can be learnt from reeler about cortical function, especially with regard to the purpose of cortical lamination. It may be possible to circumvent this problem by utilizing a recently established floxed reelin mouse (Lane-Donovan et al., 2015). For example, one may imagine a conditional reelin knockout (cKO) restricting the loss of reelin during development to specific areas and cell types. Such an approach would in principle enable the creation of a mouse line in which reelin expression is lost in the cortex only, preventing the cerebellar atrophy and ensuing motor deficits as well as all other subcortical abnormalities reported that complicate the behavioral study of the reeler mouse, while preserving the lamination defects. Unfortunately, the necessary cre-driver line to achieve this high level of specificity so far does not seem to be available. An alternative approach would be to design a cKO animal in which reelin expression is lost in adulthood only, leaving the process of cortical lamination unchanged. By comparing the behavioral phenotype in cortex-dependent tasks of such a cKO mouse with that of the reeler mouse, one may disentangle which aspects of the reeler phenotype are due to lamination defects and which are caused by the loss of the well documented role of reelin in regulating synaptic transmission in the adult brain. Indeed, a phenotype observed in the reeler mutant only but not in cKO animals can be safely assumed to relate to abnormal lamination, while a phenotype shared by both lines is more likely to result from impairments in synaptic modulation. Such an approach was recently used by Lane-Donovan et al. (2015), who generated a reelin cKO mouse that allows for tamoxifen-induced, cre-dependent suppression of reelin expression in normal, fully grown animals. The cKO mouse showed normal lamination of the hippocampus, suggesting that brain development is indeed intact. The density of spines along dendrites of individual hippocampal neurons was also unchanged in cKO mice with respect to control animals receiving vehicle injections, indicating that the reduction in spine density observed in reeler hippocampus may relate to developmental defects rather than reelin dependent spine plasticity in the adult brain (Niu et al., 2008; Lane-Donovan et al., 2015). Conversely, the cKO line exhibits slightly reduced anxiety levels when tested in the open field paradigm (Lane-Donovan et al., 2015), a trait they share with reeler animals (Salinger et al., 2003) and is probably related to the roles of reelin in the adult brain rather than to developmental defects. To our knowledge, no study to date has compared the performance of sensory systems between reeler and reelin cKO animals, but we believe that such approaches hold great promise in solving the conundrum mentioned above. In summary, although the reeler model has limitations that will hopefully be overcome in the near future, we still believe that it largely supports our conclusion that layers do not have essential computational functions.

## A Glimpse into Bird Pallium as A Non-Laminated Cortex-Like Structure

**Figure 4:**
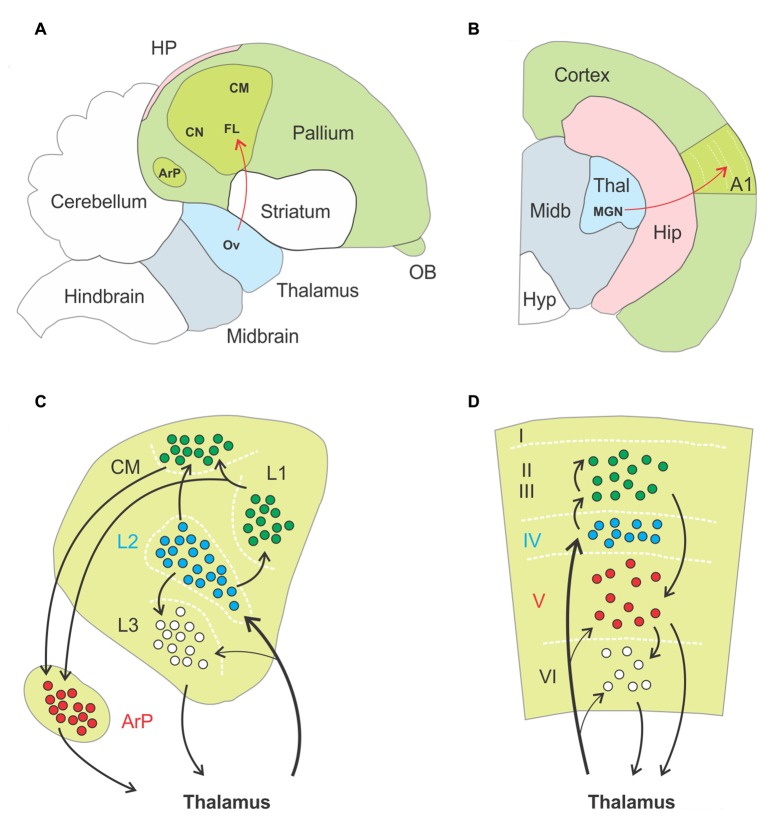
Anatomo-functional homologies between avian and mammalian brain. **(A,B)** Schematic drawings of a parasagittal section through the brain of a zebra finch, **(A**, adapted from Jarvis et al., 2005) and a coronal section through the hemisphere of a mouse **(B)**, respectively. Basic anatomical compartments present on both schematic drawings are color coded (as opposed to white). The primary auditory areas are highlighted in apple green as an example of functional homologies between both species, ascending thalamopallial and thalamocortical pathways indicated in red. Abbreviations: A1, primary auditory cortex; ArP, arcopallium; CM, caudal mesopallium; CN, caudal nidopallium; FL, field L of the nidopallium; HP, hippocampal complex; Hip, hippocampus; Hyp, hypothalamus; MGN, medial geniculate nucleus; Midb, midbrain; OB, olfactory bulb; Ov, nucleus ovoidalis; Thal, thalamus. **(C,D)** Schematic drawings of the functional organization of the auditory pallium **(C)** and primary auditory cortex **(D)**, respectively. Black arrows indicate excitatory connections, dashed white lines highlight approximate borders between pallial nuclei **(C)** and cortical layers **(D)**. Roman numerals label individual layers. Proposed homologies between discrete nuclei and layers are color coded. Thalamorecipient, RORß expressing neurons are labeled in blue, and projection neurons positive for ER81 in red. In both species these populations are linked by intermediate excitatory neurons located more superficially, in subfield L and the caudal mesopallium in birds and in supragranular layers in rodents. Abbreviations: ArP, arcopallium; CM, caudal mesopallium; L1, L2, L3, subfields L1, L2, L3 of the nidopallium.

The reeler mouse is not the only relevant model available, so let us briefly turn to birds. Bird brains lack a laminated neocortex entirely, and for this reason were once thought to be incapable of the finer perceptual and cognitive skills of mammals. Such a view has largely evolved, however, given that some birds in fact possess cognitive abilities that rival those of mammals, including, beyond the obvious capacity for complex social communication: tool use and manufacture (Kenward et al., 2005), abstract numerical skills (Scarf et al., 2011; Ditz and Nieder, 2015), capacity for causal reasoning (Taylor et al., 2012), and anticipation of the future (Clayton et al., 2003; Raby et al., 2007). The fact that birds have cognitive abilities that match those of mammals suggests that mammalian and avian brains must conduct similar operations, in spite of a different organization. The seat of the more advanced capacities of birds is thought to be the pallium, a somewhat cortex-like mantle covering the basal ganglia. For instance, two avian pallial structures, the Wulst and the dorsal ventricular ridge (DVR) were proposed as the avian homolog of the sensory neocortex (Jarvis et al., 2005; Reiner et al., 2005; Butler and Cotterill, 2006). Like the neocortex, the avian pallium exhibits areal functional specialization and receives ascending sensory information from the thalamus (Reiner et al., 2005). Unlike the neocortex, the avian pallium is organized as a set of contiguous nuclei, but remarkable homologies between nuclei and cortical layers were observed (Figure 4).

First, thalamorecipient, excitatory interneurons and projection neurons are spatially segregated in the sensory pallium. As an example, the auditory region of the pallium comprises the field L of the nidopallium, the caudal mesopallium and the arcopallium. Field L is subdivided in three subfields named L1, L2 and L3. Thalamorecipient neurons are found primarily in L2 and project to subfields L1, L3 and the caudal mesopallium. These areas are composed of excitatory interneurons, while brainstem projection neurons are located in the arcopallium (Karten, 1997; Jarvis et al., 2005). This mirrors to some extent the segregation of neurons into layers in the neocortex, where thalamorecipient “excitatory interneurons” dwell in layer IV, “intratelencephalic projection neurons” in layer II/III and “subcerebral projection neurons” or “pyramidal tract neurons” in the infragranular layers (Harris and Shepherd, 2015). Second, thalamorecipient and projection neurons in birds can be discriminated on the basis their gene expression pattern, with strong homologies to mammalian neocortex. For instance, thalamorecipient neurons of the auditory pallium express the marker gene RORB, which is also enriched in layer IV of the neocortex, whereas the marker gene ER81 identifies projection neurons in both the avian arcopallium and mammalian layer V (Boyle et al., 2011; Dugas-Ford et al., 2012; Wagener et al., 2016). Third, the spread of sensory information in the avian auditory pallium follows a temporal structure similar to what occurs in a cortical column. Thalamorecipient neurons in field L2 respond with shortest latencies to sensory input, followed by neurons in field L3 and then neurons in field L1 and in the caudal mesopallium; responses in the secondary auditory pallium appear last (Calabrese and Woolley, 2015). This sequence of events matches that of the neocortex, where thalamorecipient neurons of LIV distribute thalamic input to other layers along the canonical microcircuit.

On the basis of these homologies, one is tempted to conclude that avian and mammalian brains possess similar sensory circuits. In fact, a long standing hypothesis is that birds and mammal independently evolved homologous brain structures endowing them with similar sensorimotor and cognitive capabilities, in a stunning example of convergent evolution (Karten, 1997, 2013; Veit and Nieder, 2013; Ditz and Nieder, 2015). Because the most obvious difference here is laminar vs. nuclear organization, the lesson for us to draw from birds is clear: cortical layers are not required for circuits to perform a refined function.

## Alternative Functions for Layers

Cortical lamination is a conserved trait across mammalian species. If layers do not participate in cortical processing, what could be their function, if they are not a mere by-product of cortical development (Rakic, 2007)? We know from the reeler model that they do little to help establish specific connections between neuronal populations. Another thought is that they may help optimize synaptic transmission between cell populations. For instance, grouping neurons in layers has the potential advantage of keeping the path length between populations that need to be connected relatively constant. A stable path length ensures synchronous transmission across many synapses, facilitating temporal summation in the postsynaptic population. The cellular dispersion in the reeler cortex may lead to a more variable average path length and thus a higher temporal jitter in synaptic transmission. Assuming a mean axon conduction velocity of 1.3 m/s in cortical neurons (Swadlow, 1989), an increase in path length of 1000 μm would add nearly a millisecond (0.77 ms) to the total conduction delay. If the neighboring neuron saw its axon shortened by the same distance, a delay of more than 1.5 ms would be introduced between the activation of their synapses, provided they fire synchronously. Such a jitter might appear small at first, but if repeated at every successive synapse along the canonical circuit, could perhaps compromise the synchrony of the entire network. To our knowledge, no data from the reeler neocortex exists that could corroborate this speculation, but it is worth noting that Kowalski et al. (2010) have described an abnormally large temporal jitter in the firing of hippocampal mossy cells in response to a stimulation of the perforant path in reeler. Another possibility is that the precise arrangement of neurons into layers represents a form of optimal solution to the problem of building a highly interconnected network within a limited volume and at a reasonable metabolic cost. The principle that neuronal placement is determined so as to minimize wiring length and space usage without compromising connectivity was initially formulated by Ramon y Cajal. It was since put to the test in quite a number of elegant studies, which showed how this principle can explain the relative positions of cortical areas (Klyachko and Stevens, 2003), the layout of neurons (Chen et al., 2006), the fraction of gray matter volume allotted to dendrites and axons (Chklovskii et al., 2002), and even aspects of neuronal morphology (Chklovskii et al., 2004). Could cortical layers have evolved as an efficient answer to similar challenges? If such an assumption is true, it leads to an interesting prediction about the reeler cortex. If lamination represents an optimal layout of neurons, it follows that the reeler cortex has a suboptimal arrangement, meaning that less space is available to fit the same elements. As a result, it seems likely that less space can be allocated to at least one component of the gray matter, be it cell bodies, neuropil, fibers, glia, or blood vessels, although it seems logical that the elements that develop latest, such as myelin sheaths, would be most affected. To our knowledge, no systematic studies have ever tested such a prediction in sufficient detail, but on first approximation, no obvious difference was reported in the number of oligodendrocytes and astrocytes (Ghandour et al., 1981; Tan et al., 2009), or in the density of blood vessels (Stubbs et al., 2009; Guy et al., 2015). Neurons may provide part of the answer: although their numbers are not significantly changed in reeler, late born neurons, which adopt the compact morphology of supragranular neurons, are overrepresented with respect to early born, large pyramidal neurons (Polleux et al., 1998; Wagener et al., 2016). Determining whether and how cellular dispersion affects the relative space allotted to various components of the gray matter in the reeler brain could shed further light on the function of cortical lamination, and we are looking forward to seeing such studies in the future.

## Concluding Remarks

Whatever the real function of cortical lamination is, the current state of our knowledge is clear: in the span of over 160 years of science, little solid positive evidence that layers participate in cortical computation has emerged, while evidence to the contrary has accumulated. The evidence presented here suggests that the function does not reside in the layer but in the circuit, irrespective of its specific spatial layout (Ye et al., 2016). Although this fact is hardly controversial, we feel that a pervasive ambiguity exists when dealing with layers, in the sense that one can easily, for the sake of convenience, use the terms of “circuits” and “layers” interchangeably. As a result, a function which is in fact carried by a circuit is slowly, by semantic shift, assigned to a layer. A classic example of this is the following statement, now commonplace in the literature: “layer IV is the primary thalamocortical input layer and starts conscious perception of sensory stimuli”. While not technically incorrect, the statement is a gross simplification. After all, layer IV is crossed by the dendrites of most pyramidal neurons dwelling elsewhere in the cortical column, so that thalamocortical input is by no means restricted to those neurons whose soma sits there. In addition, excitatory neurons may quickly redistribute input from the thalamus by means of their local axonal collaterals, so that cortical activity nearly instantaneously spreads over several layers and columns to mediate perception of sensory stimuli (Reyes-Puerta et al., 2015). Thus, simplifications such as these can be confusing and quite unhelpful, and we would like to urge us all to use a clear language when writing about layers, so as to not give them functions they do not have.

## Author Contributions

JFS and JG: conception and drafting of the manuscript; conception and generation of figures.

## Conflict of Interest Statement

The authors declare that the research was conducted in the absence of any commercial or financial relationships that could be construed as a potential conflict of interest.
